# Editorial: Non-thermal Technologies

**DOI:** 10.3389/fnut.2021.752799

**Published:** 2021-08-26

**Authors:** Fabiano A. N. Fernandes, Antonio Morata, Sueli Rodrigues

**Affiliations:** ^1^Departamento de Engenharia Química, Universidade Federal Do Ceará, Fortaleza, Brazil; ^2^enotecUPM, Universidad Politécnica de Madrid, Madrid, Spain; ^3^Departamento de Engenharia de Alimentos, Universidade Federal Do Ceará, Fortaleza, Brazil

**Keywords:** non-thermal, food processing, food quality and safety, emerging technologies, food science and technology

Non-thermal technologies for food applications comprises technologies such as ultrasound, cold plasma, high-pressure, pulsed electric field, ozone, UV, electrolyzed water, and pulsed UV light. The development of these technologies has been growing in the last decade due to its successful application in sanitization, but also as processes that can improve nutritional and sensory properties of food products. Some technologies, such as high-pressure processing, ozone, UV light, and pulsed electric field have been used in industrial processes for at least two decades. While others, such as ultrasound, cold plasma, pulsed UV light are still under development, and are currently in lab- and pilot-scale phases.

Pasteurization, sterilization, and other thermal technologies used to sanitize food products usually affects negatively the nutritional and sensory properties of many products. As food quality and nutrition becomes a major concern in the modern world, it is not possible anymore to rely on technologies that sanitize our food but that also reduce their sensory and nutritional quality. Studies with non-thermal technologies, at first, aimed at achieving the same level of sanitization, while maintaining the nutritional and sensory properties of the products as similar as possible to natural. The application of these non-thermal processes in sanitization has been well-explored, and the knowledge of the strains of microorganisms that can be inactivated and how this inactivation is achieved was the focus of several research. In this Research Topic, articles on ultra-high pressure homogenization by Morata and Guamis and acidic electrolyzed water by Liu et al. present some aspects involving non-thermal technology and sanitization.

Although non-thermal technologies do not involve heat, several of these technologies induce chemical reactions in the food product. These reactions may alter the food tissue structure, enzymes, metabolic rates, phenolics, antioxidants, vitamins, color, and other sensory and nutritional aspects of our food. Under some circumstances the changes are positive, improving the phenolic and free vitamin content, inactivating color degrading enzymes, improving cloud stability in juices; but there are also reports on negative effects such as color fading, texture loss and production of off-flavor. Reports on these kinds of effects of non-thermal technologies on food is still missing and are the primary focus of this Research Topic.

Articles on the impact of ultra-high pressure homogenization, plasma treated water, ultrasound on different food products can be found in this Research Topic, as well as a review on the theory and application of several non-thermal technologies. The article written by Morata and Guamis shows a good examples of how non-thermal technologies can boost the production of wines and juices with higher antioxidant capacities without resorting to the addition of sulfur dioxide. Schnabel et al. present how plasma treated water can be an effective and low-cost process to extend the shelf-life of vegetables, while Liu et al. report on the application of acidic electrolyzed water to process fish. A review on the theory and application of several non-thermal technologies is presented by Jadhav et al., while Astráin-Redín et al. focus on direct contact ultrasound.

Non-thermal technologies have also been used in non-invasive quality control, where the product or the process can be monitored. In this Research Topic, Wang et al. demonstrate the efficacy of on low-field nuclear magnetic resonance in monitoring the quality control of aquatic products.

This Research Topic illustrate some of the possibilities that non-thermal emerging technologies open in the new food technology allowing the production of safer, healthier, and more sensory-attractive foods.

## Author Contributions

FF prepared a draft concept on the present Resource Topic, which was supplemented and corrected by SR and AM. All authors contributed to the article and approved the submitted version.

## Conflict of Interest

The authors declare that the research was conducted in the absence of any commercial or financial relationships that could be construed as a potential conflict of interest.

## Publisher's Note

All claims expressed in this article are solely those of the authors and do not necessarily represent those of their affiliated organizations, or those of the publisher, the editors and the reviewers. Any product that may be evaluated in this article, or claim that may be made by its manufacturer, is not guaranteed or endorsed by the publisher.

